# (*E*)-*N*′-(4-Meth­oxy­benzyl­idene)-2-(2-methyl-4-nitro-1*H*-imidazol-1-yl)acetohydrazide

**DOI:** 10.1107/S1600536812039621

**Published:** 2012-09-22

**Authors:** Hoong-Kun Fun, Tze Shyang Chia, Priya V. Frank, Mahesha Poojary, Balakrishna Kalluraya

**Affiliations:** a X-ray Crystallography Unit, School of Physics, Universiti Sains Malaysia, 11800 USM, Penang, Malaysia; b Department of Pharmaceutical Chemistry, College of Pharmacy, King Saud University, Riyadh 11451, Saudi Arabia; c Department of Studies in Chemistry, Mangalore University, Mangalagangotri, Mangalore 574 199, India; d Department of Chemistry, Canara Engineering College, Mangalore 574 199, India

## Abstract

In the title compound, C_14_H_15_N_5_O_4_, the central –C=N—N—C(=O)—C– bridge is nearly planar [maximum deviation = 0.037 (1) Å] and forms dihedral angles of 7.37 (9) and 73.33 (5)°, respectively, with the benzene and imidazole rings. The dihedral angle between the benzene and imidazole rings is 66.08 (9)°. The meth­oxy and nitro groups are nearly coplanar with the benzene and imidazole rings, respectively, with a C—O—C—C torsion angle of 5.9 (2)° and an O—N—C—C angle of −0.2 (2)°. In the crystal, mol­ecules are linked by a pair of N—H⋯O hydrogen bonds with an *R*_2_^2^(8) ring motif, forming an inversion dimer. The dimers are further inter­connected by C—H⋯O hydrogen bonds into a sheet parallel to the (111) plane. A C—H⋯π inter­action is also observed between the sheets.

## Related literature

For applications and biological activities of imidazole derivatives, see: Frank & Kalluraya (2005 ▶); Dobler (2003 ▶); Gauthier & Duceppe (1984 ▶); Khan & Nandan (1997 ▶); Khabnadideh *et al.* (2003 ▶). For the stability of the temperature controller used for data collection, see: Cosier & Glazer (1986 ▶). For hydrogen-bond motifs, see: Bernstein *et al.* (1995 ▶).
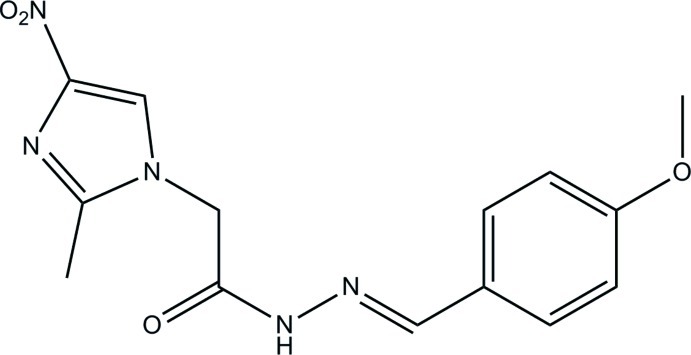


## Experimental

### 

#### Crystal data

C_14_H_15_N_5_O_4_
*M* *_r_* = 317.31
Triclinic, 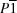

*a* = 4.3366 (1) Å
*b* = 12.9773 (3) Å
*c* = 13.2138 (3) Å
α = 84.919 (2)°
β = 87.353 (2)°
γ = 84.611 (1)°
*V* = 736.90 (3) Å^3^
*Z* = 2
Mo *K*α radiation
μ = 0.11 mm^−1^
*T* = 100 K
0.51 × 0.19 × 0.11 mm

#### Data collection

Bruker SMART APEXII CCD area-detector diffractometer
Absorption correction: multi-scan (*SADABS*; Bruker, 2009 ▶) *T*_min_ = 0.947, *T*_max_ = 0.988
15663 measured reflections
4303 independent reflections
3252 reflections with *I* > 2σ(*I*)
*R*_int_ = 0.035

#### Refinement

*R*[*F* ^2^ > 2σ(*F* ^2^)] = 0.053
*wR*(*F* ^2^) = 0.131
*S* = 1.03
4303 reflections
214 parameters
H atoms treated by a mixture of independent and constrained refinement
Δρ_max_ = 0.34 e Å^−3^
Δρ_min_ = −0.31 e Å^−3^

### 

Data collection: *APEX2* (Bruker, 2009 ▶); cell refinement: *SAINT* (Bruker, 2009 ▶); data reduction: *SAINT*; program(s) used to solve structure: *SHELXTL* (Sheldrick, 2008 ▶); program(s) used to refine structure: *SHELXTL*; molecular graphics: *SHELXTL*; software used to prepare material for publication: *SHELXTL* and *PLATON* (Spek, 2009 ▶).

## Supplementary Material

Crystal structure: contains datablock(s) global, I. DOI: 10.1107/S1600536812039621/is5195sup1.cif

Structure factors: contains datablock(s) I. DOI: 10.1107/S1600536812039621/is5195Isup2.hkl

Supplementary material file. DOI: 10.1107/S1600536812039621/is5195Isup3.cml

Additional supplementary materials:  crystallographic information; 3D view; checkCIF report

## Figures and Tables

**Table 1:** Hydrogen-bond geometry (Å, °) *Cg*1 is the centroid of the N3/C11/C12/N4/C13 ring.

*D*—H⋯*A*	*D*—H	H⋯*A*	*D*⋯*A*	*D*—H⋯*A*
N2—H1*N*2⋯O2^i^	0.88 (2)	2.06 (2)	2.9372 (17)	176 (2)
C11—H11*A*⋯O4^ii^	0.95	2.28	3.186 (2)	160
C14—H14*A*⋯O1^iii^	0.98	2.46	3.434 (2)	173
C14—H14*C*⋯*Cg*1^iv^	0.98	2.74	3.4747 (18)	133

Symmetry codes: (i) 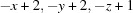; (ii) 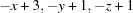; (iii) 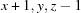; (iv) 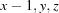.

**Fig. 1.**
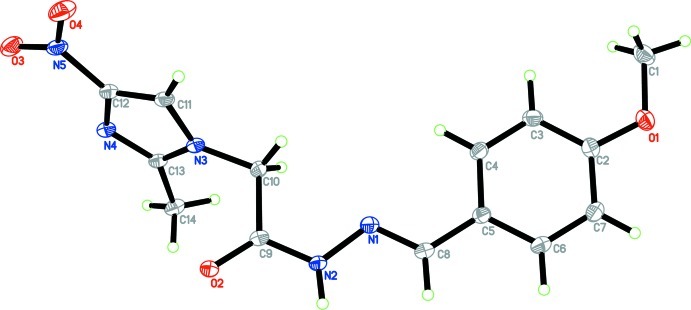
The molecular structure of the title compound with atom labels and 50% probability displacement ellipsoids.

**Fig. 2.**
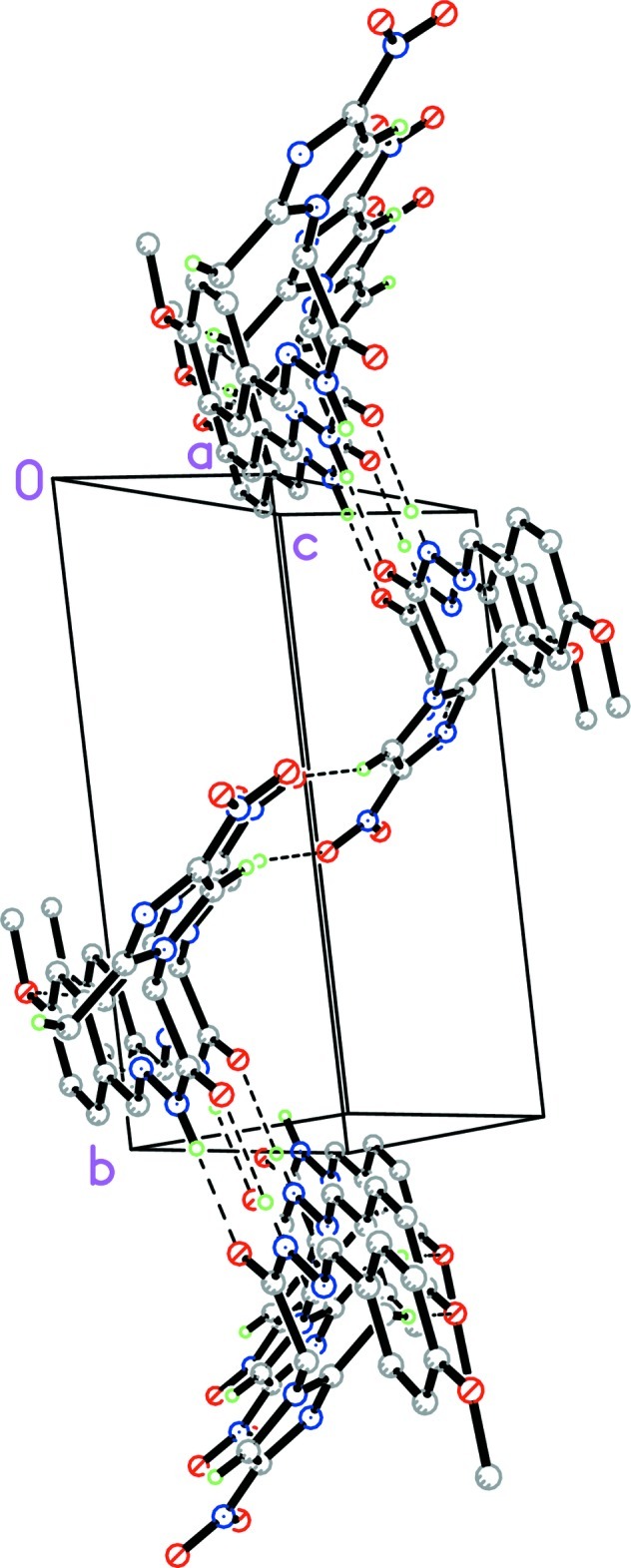
The crystal packing of the title compound viewed along the [101] axis. The dashed lines represent the hydrogen bonds. For clarity sake, hydrogen atoms not involved in hydrogen bonding have been omitted.

## supplementary crystallographic information

### Comment

Various applications of imidazoles are listed in the literature with functions as widely divergent as dyestuffs, catalysts, polymerizing agents, drugs, herbicides and fungicides (Frank & Kalluraya, 2005). Imidazole derivatives show promising antiallergic (Gauthier & Duceppe, 1984), anti-inflammatory, analgesic (Khan & Nandan, 1997) and antibacterial (Khabnadideh *et al.*, 2003) activities. Imidazole derivatives are also useful for the treatment of rheumatoid arthritis and inflammatory diseases (Dobler, 2003). In view of the apparent importance of imidazole derivatives as potential pharmacological agents, and in continuation of our research work in the field of biologically active imidazole derivatives, we report herein the crystal structure of the title compound.

The asymmetric unit of the title compound is shown in Fig. 1. The benzene (C2–C7) and imidazole (N3/C11/C12/N4/C13) rings make a dihedral angle of 66.08 (9)° with each other. The —C8═N1—N2—C9(═O2)—C10— bridge is nearly planar [maximum deviation = 0.037 (1) Å at atom N2] and forms dihedral angles of 7.37 (9) and 73.33 (5)° with the benzene and imidazole rings, respectively. The methoxy (O1/C1) and nitro (O3/O4/N5) groups are coplanar with the benzene ring and the imidazole ring, respectively, as indicated by torsion angles C1—O1—C2—C3 [5.9 (2)°], O4—N5—C12—C11 [-0.2 (2)°] and O3—N5—C12—N4 [0.0 (2)°].

In the crystal (Fig. 2), molecules are linked by a pair of intermolecular N2—H1N2···O2 hydrogen bonds into an inversion dimer with an *R*_2_^2^(8) ring motif (Bernstein *et al.*, 1995). The dimers are further interconnected by C11—H11A···O4 and C14—H14A···O1 hydrogen bonds into a sheet structure parallel to the (111) plane. The crystal is further stabilized by a C—H···π interaction (Table 1), involving *Cg*1 which is the centroid of the N3/C11/C12/N4/C13 ring.

### Experimental

The title compound was synthesized by refluxing a mixture of 2-(2-methyl-4-nitro-1*H*-imidazol-1-yl)acetohydrazide (0.1 mol) and 1-(4-methoxyphenyl)ethanone (0.1 mol) in glacial acetic acid for 1 h. On cooling the reaction mixture to room temperature and evaporation of the solvent under reduced pressure, the solid that separated out was filtered, washed with water and dried. Yellow plate-shaped crystals were grown from ethanol-dioxane mixture by slow evaporation method (m.p. 505 K).

### Refinement

The N-bound H atom was located in a difference Fourier map and refined freely [N2—H1N2 = 0.88 (2) Å]. The remaining H atoms were positioned geometrically (C—H = 0.95, 0.98 and 0.99 Å) and refined using a riding model with *U*_iso_(H) = 1.2 or 1.5*U*_eq_(C). A rotating group model was applied to the methyl group.

### Crystal data

**Table:**

C_14_H_15_N_5_O_4_	*Z* = 2
*M**_r_* = 317.31	*F*(000) = 332
Triclinic, *P*1	*D*_x_ = 1.430 Mg m^−^^3^
Hall symbol: -P 1	Mo *K*α radiation, λ = 0.71073 Å
*a* = 4.3366 (1) Å	Cell parameters from 5229 reflections
*b* = 12.9773 (3) Å	θ = 2.3–30.0°
*c* = 13.2138 (3) Å	µ = 0.11 mm^−^^1^
α = 84.919 (2)°	*T* = 100 K
β = 87.353 (2)°	Plate, yellow
γ = 84.611 (1)°	0.51 × 0.19 × 0.11 mm
*V* = 736.90 (3) Å^3^	

### Data collection

**Table:**

Bruker SMART APEXII CCD area-detector diffractometer	4303 independent reflections
Radiation source: fine-focus sealed tube	3252 reflections with *I* > 2σ(*I*)
Graphite monochromator	*R*_int_ = 0.035
φ and ω scans	θ_max_ = 30.1°, θ_min_ = 1.6°
Absorption correction: multi-scan (*SADABS*; Bruker, 2009)	*h* = −6→6
*T*_min_ = 0.947, *T*_max_ = 0.988	*k* = −18→18
15663 measured reflections	*l* = −18→18

### Refinement

**Table:**

Refinement on *F*^2^	Primary atom site location: structure-invariant direct methods
Least-squares matrix: full	Secondary atom site location: difference Fourier map
*R*[*F*^2^ > 2σ(*F*^2^)] = 0.053	Hydrogen site location: inferred from neighbouring sites
*wR*(*F*^2^) = 0.131	H atoms treated by a mixture of independent and constrained refinement
*S* = 1.03	*w* = 1/[σ^2^(*F*_o_^2^) + (0.0518*P*)^2^ + 0.496*P*] where *P* = (*F*_o_^2^ + 2*F*_c_^2^)/3
4303 reflections	(Δ/σ)_max_ < 0.001
214 parameters	Δρ_max_ = 0.34 e Å^−^^3^
0 restraints	Δρ_min_ = −0.31 e Å^−^^3^

### Special details

**Table:**

Experimental. The crystal was placed in the cold stream of an Oxford Cryosystems Cobra open-flow nitrogen cryostat (Cosier & Glazer, 1986) operating at 100.0 (1) K.
Geometry. All e.s.d.'s (except the e.s.d. in the dihedral angle between two l.s. planes) are estimated using the full covariance matrix. The cell e.s.d.'s are taken into account individually in the estimation of e.s.d.'s in distances, angles and torsion angles; correlations between e.s.d.'s in cell parameters are only used when they are defined by crystal symmetry. An approximate (isotropic) treatment of cell e.s.d.'s is used for estimating e.s.d.'s involving l.s. planes.
Refinement. Refinement of *F*^2^ against ALL reflections. The weighted *R*-factor *wR* and goodness of fit *S* are based on *F*^2^, conventional *R*-factors *R* are based on *F*, with *F* set to zero for negative *F*^2^. The threshold expression of *F*^2^ > σ(*F*^2^) is used only for calculating *R*-factors(gt) *etc*. and is not relevant to the choice of reflections for refinement. *R*-factors based on *F*^2^ are statistically about twice as large as those based on *F*, and *R*- factors based on ALL data will be even larger.

### Fractional atomic coordinates and isotropic or equivalent isotropic displacement parameters (Å^2^)

**Table:**

	*x*	*y*	*z*	*U*_iso_*/*U*_eq_	
O1	−0.4105 (3)	0.76565 (9)	1.00477 (9)	0.0228 (3)	
O2	1.0816 (3)	0.88398 (8)	0.42690 (8)	0.0168 (2)	
O3	1.4713 (3)	0.47331 (9)	0.20227 (9)	0.0237 (3)	
O4	1.5848 (3)	0.44149 (9)	0.36131 (9)	0.0256 (3)	
N1	0.5223 (3)	0.85992 (9)	0.62488 (10)	0.0157 (3)	
N2	0.7370 (3)	0.90552 (10)	0.55866 (10)	0.0151 (3)	
N3	0.9336 (3)	0.68443 (9)	0.40406 (10)	0.0156 (3)	
N4	1.0435 (3)	0.63333 (9)	0.24863 (10)	0.0164 (3)	
N5	1.4374 (3)	0.49076 (9)	0.29156 (10)	0.0175 (3)	
C1	−0.4567 (4)	0.65710 (13)	1.02022 (13)	0.0243 (4)	
H1A	−0.6064	0.6458	1.0771	0.036*	
H1B	−0.2590	0.6171	1.0357	0.036*	
H1C	−0.5361	0.6344	0.9584	0.036*	
C2	−0.2144 (4)	0.79626 (12)	0.92693 (12)	0.0178 (3)	
C3	−0.0763 (4)	0.73140 (12)	0.85563 (12)	0.0181 (3)	
H3A	−0.1178	0.6605	0.8593	0.022*	
C4	0.1213 (4)	0.77065 (11)	0.77954 (12)	0.0164 (3)	
H4A	0.2139	0.7261	0.7311	0.020*	
C5	0.1872 (4)	0.87453 (11)	0.77251 (11)	0.0149 (3)	
C6	0.0467 (4)	0.93826 (12)	0.84475 (12)	0.0193 (3)	
H6A	0.0893	1.0090	0.8415	0.023*	
C7	−0.1527 (4)	0.90054 (12)	0.92063 (12)	0.0204 (3)	
H7A	−0.2477	0.9453	0.9685	0.025*	
C8	0.4071 (4)	0.91518 (11)	0.69559 (12)	0.0155 (3)	
H8A	0.4658	0.9836	0.6979	0.019*	
C9	0.8830 (4)	0.84969 (11)	0.48733 (11)	0.0141 (3)	
C10	0.7805 (4)	0.74033 (11)	0.48597 (12)	0.0172 (3)	
H10A	0.8265	0.7006	0.5518	0.021*	
H10B	0.5534	0.7452	0.4782	0.021*	
C11	1.1547 (4)	0.60235 (11)	0.41596 (12)	0.0162 (3)	
H11A	1.2447	0.5722	0.4771	0.019*	
C12	1.2159 (4)	0.57381 (11)	0.31963 (12)	0.0156 (3)	
C13	0.8741 (4)	0.70062 (11)	0.30221 (12)	0.0156 (3)	
C14	0.6539 (4)	0.78659 (12)	0.26004 (13)	0.0197 (3)	
H14A	0.6200	0.7773	0.1887	0.030*	
H14B	0.7403	0.8531	0.2645	0.030*	
H14C	0.4563	0.7861	0.2991	0.030*	
H1N2	0.792 (5)	0.9681 (18)	0.5660 (16)	0.031 (6)*	

### Atomic displacement parameters (Å^2^)

**Table:**

	*U*^11^	*U*^22^	*U*^33^	*U*^12^	*U*^13^	*U*^23^
O1	0.0275 (7)	0.0223 (6)	0.0192 (6)	−0.0081 (5)	0.0062 (5)	−0.0019 (4)
O2	0.0186 (6)	0.0126 (5)	0.0197 (5)	−0.0047 (4)	0.0036 (4)	−0.0028 (4)
O3	0.0346 (7)	0.0162 (5)	0.0199 (6)	0.0018 (5)	0.0016 (5)	−0.0055 (4)
O4	0.0341 (7)	0.0176 (5)	0.0242 (6)	0.0067 (5)	−0.0071 (5)	−0.0021 (5)
N1	0.0165 (6)	0.0127 (5)	0.0178 (6)	−0.0019 (5)	0.0009 (5)	−0.0012 (5)
N2	0.0173 (7)	0.0104 (5)	0.0181 (6)	−0.0040 (5)	0.0031 (5)	−0.0033 (5)
N3	0.0200 (7)	0.0099 (5)	0.0174 (6)	−0.0032 (5)	0.0016 (5)	−0.0034 (4)
N4	0.0195 (7)	0.0113 (5)	0.0185 (6)	−0.0022 (5)	0.0003 (5)	−0.0023 (5)
N5	0.0228 (7)	0.0096 (5)	0.0204 (7)	−0.0017 (5)	−0.0009 (5)	−0.0025 (5)
C1	0.0275 (9)	0.0232 (8)	0.0224 (8)	−0.0092 (7)	0.0021 (7)	0.0024 (6)
C2	0.0183 (8)	0.0197 (7)	0.0155 (7)	−0.0027 (6)	0.0003 (6)	−0.0007 (6)
C3	0.0219 (8)	0.0135 (6)	0.0191 (7)	−0.0031 (6)	−0.0011 (6)	−0.0009 (5)
C4	0.0175 (8)	0.0140 (6)	0.0179 (7)	0.0000 (6)	0.0002 (6)	−0.0034 (5)
C5	0.0151 (7)	0.0128 (6)	0.0168 (7)	−0.0007 (6)	−0.0005 (6)	−0.0020 (5)
C6	0.0232 (8)	0.0131 (6)	0.0221 (8)	−0.0024 (6)	0.0008 (7)	−0.0045 (6)
C7	0.0231 (8)	0.0182 (7)	0.0204 (8)	−0.0022 (6)	0.0040 (7)	−0.0065 (6)
C8	0.0173 (7)	0.0112 (6)	0.0182 (7)	−0.0009 (6)	−0.0017 (6)	−0.0021 (5)
C9	0.0153 (7)	0.0111 (6)	0.0163 (7)	−0.0014 (5)	−0.0019 (6)	−0.0022 (5)
C10	0.0223 (8)	0.0128 (6)	0.0173 (7)	−0.0046 (6)	0.0050 (6)	−0.0047 (5)
C11	0.0205 (8)	0.0101 (6)	0.0185 (7)	−0.0034 (6)	−0.0003 (6)	−0.0015 (5)
C12	0.0178 (8)	0.0102 (6)	0.0190 (7)	−0.0023 (6)	0.0009 (6)	−0.0017 (5)
C13	0.0188 (8)	0.0096 (6)	0.0191 (7)	−0.0035 (6)	0.0002 (6)	−0.0029 (5)
C14	0.0229 (8)	0.0130 (6)	0.0229 (8)	0.0016 (6)	−0.0023 (7)	−0.0025 (6)

### Geometric parameters (Å, º)

**Table:**

O1—C2	1.3599 (19)	C3—C4	1.384 (2)
O1—C1	1.437 (2)	C3—H3A	0.9500
O2—C9	1.2340 (18)	C4—C5	1.398 (2)
O3—N5	1.2206 (17)	C4—H4A	0.9500
O4—N5	1.2410 (18)	C5—C6	1.399 (2)
N1—C8	1.2825 (19)	C5—C8	1.460 (2)
N1—N2	1.3853 (17)	C6—C7	1.380 (2)
N2—C9	1.3394 (19)	C6—H6A	0.9500
N2—H1N2	0.88 (2)	C7—H7A	0.9500
N3—C11	1.368 (2)	C8—H8A	0.9500
N3—C13	1.376 (2)	C9—C10	1.5284 (19)
N3—C10	1.4565 (19)	C10—H10A	0.9900
N4—C13	1.3208 (19)	C10—H10B	0.9900
N4—C12	1.365 (2)	C11—C12	1.364 (2)
N5—C12	1.4371 (19)	C11—H11A	0.9500
C1—H1A	0.9800	C13—C14	1.485 (2)
C1—H1B	0.9800	C14—H14A	0.9800
C1—H1C	0.9800	C14—H14B	0.9800
C2—C3	1.396 (2)	C14—H14C	0.9800
C2—C7	1.399 (2)		
			
C2—O1—C1	117.64 (13)	C5—C6—H6A	119.3
C8—N1—N2	115.67 (12)	C6—C7—C2	119.82 (14)
C9—N2—N1	118.95 (12)	C6—C7—H7A	120.1
C9—N2—H1N2	119.3 (14)	C2—C7—H7A	120.1
N1—N2—H1N2	121.5 (14)	N1—C8—C5	121.01 (13)
C11—N3—C13	107.62 (12)	N1—C8—H8A	119.5
C11—N3—C10	125.49 (13)	C5—C8—H8A	119.5
C13—N3—C10	126.83 (13)	O2—C9—N2	122.86 (13)
C13—N4—C12	103.74 (13)	O2—C9—C10	122.67 (13)
O3—N5—O4	124.20 (14)	N2—C9—C10	114.47 (13)
O3—N5—C12	119.08 (13)	N3—C10—C9	112.56 (12)
O4—N5—C12	116.72 (13)	N3—C10—H10A	109.1
O1—C1—H1A	109.5	C9—C10—H10A	109.1
O1—C1—H1B	109.5	N3—C10—H10B	109.1
H1A—C1—H1B	109.5	C9—C10—H10B	109.1
O1—C1—H1C	109.5	H10A—C10—H10B	107.8
H1A—C1—H1C	109.5	C12—C11—N3	103.93 (14)
H1B—C1—H1C	109.5	C12—C11—H11A	128.0
O1—C2—C3	124.58 (14)	N3—C11—H11A	128.0
O1—C2—C7	115.76 (14)	C11—C12—N4	113.11 (14)
C3—C2—C7	119.66 (14)	C11—C12—N5	125.50 (14)
C4—C3—C2	119.79 (14)	N4—C12—N5	121.40 (13)
C4—C3—H3A	120.1	N4—C13—N3	111.59 (14)
C2—C3—H3A	120.1	N4—C13—C14	125.55 (14)
C3—C4—C5	121.31 (14)	N3—C13—C14	122.82 (13)
C3—C4—H4A	119.3	C13—C14—H14A	109.5
C5—C4—H4A	119.3	C13—C14—H14B	109.5
C4—C5—C6	118.03 (14)	H14A—C14—H14B	109.5
C4—C5—C8	121.61 (13)	C13—C14—H14C	109.5
C6—C5—C8	120.30 (13)	H14A—C14—H14C	109.5
C7—C6—C5	121.39 (14)	H14B—C14—H14C	109.5
C7—C6—H6A	119.3		
			
C8—N1—N2—C9	175.35 (14)	C13—N3—C10—C9	−74.30 (19)
C1—O1—C2—C3	5.9 (2)	O2—C9—C10—N3	−2.2 (2)
C1—O1—C2—C7	−174.01 (15)	N2—C9—C10—N3	177.07 (14)
O1—C2—C3—C4	−179.73 (15)	C13—N3—C11—C12	0.07 (16)
C7—C2—C3—C4	0.2 (3)	C10—N3—C11—C12	177.30 (13)
C2—C3—C4—C5	0.2 (2)	N3—C11—C12—N4	−0.37 (17)
C3—C4—C5—C6	−0.2 (2)	N3—C11—C12—N5	179.33 (13)
C3—C4—C5—C8	176.91 (15)	C13—N4—C12—C11	0.52 (17)
C4—C5—C6—C7	−0.3 (2)	C13—N4—C12—N5	−179.19 (13)
C8—C5—C6—C7	−177.45 (16)	O3—N5—C12—C11	−179.70 (15)
C5—C6—C7—C2	0.8 (3)	O4—N5—C12—C11	−0.2 (2)
O1—C2—C7—C6	179.25 (16)	O3—N5—C12—N4	0.0 (2)
C3—C2—C7—C6	−0.7 (3)	O4—N5—C12—N4	179.49 (14)
N2—N1—C8—C5	−177.68 (14)	C12—N4—C13—N3	−0.46 (16)
C4—C5—C8—N1	7.6 (2)	C12—N4—C13—C14	177.23 (15)
C6—C5—C8—N1	−175.37 (15)	C11—N3—C13—N4	0.26 (17)
N1—N2—C9—O2	−178.50 (14)	C10—N3—C13—N4	−176.93 (13)
N1—N2—C9—C10	2.3 (2)	C11—N3—C13—C14	−177.51 (14)
C11—N3—C10—C9	108.99 (16)	C10—N3—C13—C14	5.3 (2)

### Hydrogen-bond geometry (Å, º)

*Cg*1 is the centroid of the N3/C11/C12/N4/C13 ring.

**Table:**

*D*—H···*A*	*D*—H	H···*A*	*D*···*A*	*D*—H···*A*
N2—H1*N*2···O2^i^	0.88 (2)	2.06 (2)	2.9372 (17)	176 (2)
C11—H11*A*···O4^ii^	0.95	2.28	3.186 (2)	160
C14—H14*A*···O1^iii^	0.98	2.46	3.434 (2)	173
C14—H14*C*···*Cg*1^iv^	0.98	2.74	3.4747 (18)	133

Symmetry codes: (i) −*x*+2, −*y*+2, −*z*+1; (ii) −*x*+3, −*y*+1, −*z*+1; (iii) *x*+1, *y*, *z*−1; (iv) *x*−1, *y*, *z*.
